# A Bumpy Ride on the Diagnostic Bench of Massive Parallel Sequencing, the Case of the Mitochondrial Genome

**DOI:** 10.1371/journal.pone.0112950

**Published:** 2014-11-10

**Authors:** Kim Vancampenhout, Ben Caljon, Claudia Spits, Katrien Stouffs, An Jonckheere, Linda De Meirleir, Willy Lissens, Arnaud Vanlander, Joél Smet, Boel De Paepe, Rudy Van Coster, Sara Seneca

**Affiliations:** 1 Research Group Reproduction and Genetics (REGE), Vrije Universiteit Brussel (VUB), Brussels, Belgium; 2 Center for Medical Genetics, UZ Brussel, Vrije Universiteit Brussel (VUB), Brussels, Belgium; 3 Department of Pediatric Neurology, UZ Brussel, Vrije Universiteit Brussel (VUB), Brussels, Belgium; 4 Department of Pediatrics, Division of Pediatric Neurology and Metabolism, University Hospital Ghent, Ghent University, Ghent, Belgium; Newcastle University, United Kingdom

## Abstract

The advent of massive parallel sequencing (MPS) has revolutionized the field of human molecular genetics, including the diagnostic study of mitochondrial (mt) DNA dysfunction. The analysis of the complete mitochondrial genome using MPS platforms is now common and will soon outrun conventional sequencing. However, the development of a robust and reliable protocol is rather challenging. A previous pilot study for the re-sequencing of human mtDNA revealed an uneven coverage, affecting predominantly part of the plus strand. In an attempt to address this problem, we undertook a comparative study of standard and modified protocols for the Ion Torrent PGM system. We could not improve strand representation by altering the recommended shearing methodology of the standard workflow or omitting the DNA polymerase amplification step from the library construction process. However, we were able to associate coverage bias of the plus strand with a specific sequence motif. Additionally, we compared coverage and variant calling across technologies. The same samples were also sequenced on a MiSeq device which showed that coverage and heteroplasmic variant calling were much improved.

## Introduction

The human mitochondrial DNA (mtDNA) is a small circular double stranded molecule that comprises 16569 bp and codes for 13 protein genes, 22 tRNAs and 2 rRNAs. All these are essential elements to the correct function of the oxidative phosphorylation (OXPHOS) system, a fundamental process of the cellular role of mitochondria. For over 25 years, the pathogenicity of certain alterations of the mitochondrial genome has been clearly established in mtDNA disease. Despite the existence of mutation hotspot genes and regions, and the occurrence of recurrent mutations, these pathogenic aberrations are scattered over the entire mitochondrial genome. This makes it necessary to completely analyze this small genome to confirm or exclude pathogenic mtDNA changes. Molecular analysis often requires different and complementary methods, e.g. Southern blot, long range (LR)-PCR, Denaturing Gradient Gel Electrophoresis (DGGE), High Resolution Melting (HRM), quantitative (q)PCR and Sanger sequencing for the detection and quantification of mtDNA. The emergence of MPS technologies has provided the diagnostic bench with a new and highly valuable tool for the evaluation of human mtDNA integrity. However, these new sequencing platforms have pitfalls, and crucial biases might be created [1] such as the loss of coverage in regions with GC-extreme (high or low) content, or the limited ability to analyze homopolymeric stretches [2] [3]. As a result, heteroplasmic variant calling might be severely complicated or even erroneous, as the nucleotide representation can be too weak or unreliable in some of these regions. In a recent study by Seneca et al. [4], the mitochondrial genomes of 32 DNA samples were analyzed using an Ion Torrent PGM system after enrichment with LR-PCR amplification of the mtDNA. A major bias in read depth between the positive and negative strand was seen for almost 10% of the mitochondrial genome, despite the fact that the sequencing was carried out at an average coverage of 6000. Moreover, in some regions the data for the positive strand dropped severely, reaching a critically low coverage. This difference in read depth between both strands made it challenging to distinguish true low-level heteroplasmic variants from sequencing errors. Therefore, we tried to develop an improved MPS-based protocol for the analysis of the human mitochondrial genome. Several library preparation methods and sequencing technologies were tested in order to ameliorate the present sequencing protocol, and their outputs were compared. We were also able to identify the specific nature of the systematically undercovered nucleotide motifs. We are convinced that our findings are of interest to all laboratories working on MPS for the mtDNA, both in a research or clinical setting.

## Materials and Methods

### Ethics Statement

This study was approved by the ethics committee of the Institutional Review Board (IRB) of the University Hospital (UZ Brussel, Vrije Universiteit Brussel). For all control samples a written informed consent was obtained. The informed consent form was also reviewed and approved by the local ethics committee of the IRB. For the patient samples, during clinical consultation oral consent was given to study their genetic material by any methods relevant to diagnostically confirm or rule out mutations in their mtDNA. This procedure does not require a written consent by the patient, and oral consent is recorded in a protected medical patient file. This is a standard procedure that is approved within the Center for Medical Genetics and accepted by the ethics committee of the IRB of the hospital.

### Sample collection and DNA

Six DNA samples, corresponding to three controls (samples 1, 2, 4 in [4]) and three patients (samples 9, 14, 21 in [4]), were randomly selected from the previous sample cohort [4]. Total DNA had been extracted from leukocytes using standard DNA isolation techniques (Chemagen, Perkin Elmer, Zaventem, Belgium). An overview of the samples and techniques used is given in Supporting Information S1.

### Long range PCR

MPS data files, obtained from a previous study, were mainly generated by the sequencing of three overlapping LR-PCR fragments covering the whole mitochondrial genome (all six samples were amplified using the ‘three overlapping’ fragment approach, two were additionally generated with a ‘single fragment’ method) [4]. However, as was demonstrated in a previous study, one large single LR-PCR product allowed the detection of variants, indels and large deletions simultaneously, a situation that is advantageous due to time and cost constrains for clinical genetic testing. For this single LR-PCR a 16.2 kb fragment [5] was generated using the LongAmp *Taq* PCR kit (New England Biolabs, Bioke, Leiden, The Netherlands). The mitochondrial genome was amplified from 200 ng gDNA as template in a 50 µL PCR assay according to manufacturer’s recommendations. The PCR protocol was adapted to an initial 30 s denaturation at 94°C, followed by 15 cycles with first a denaturation of 10 s at 92°C, annealing at 67°C for 30 s and an extension of 10 min at 68°C. This was followed by 18 cycles with a denaturation of 10 s at 92°C and an extension of 10 min +20 s every cycle at 68°C. A final extension step was performed at 68°C for 7 min. Successful PCR amplification was assessed using 0.8% agarose gel electrophoresis, and products were purified with AMpure beads (Analis, Champion, Belgium).

### Ion Torrent PGM sequencing

Ion Torrent semi-conductor sequencing technology detects the incorporation of each of the four nucleotides as small changes in pH that are provoked by the release of a proton. Library and template preparation include an amplification step. The latter is known as an emulsion PCR which takes place in aqueous droplets suspended in oil.

The data files of six samples, previously sequenced using the Ion Torrent PGM assay according to the manufacturer’s instructions [4], were regarded as benchmark material for a comparative study of the new protocols described in the present study. We evaluated the following modifications to the standard protocol: different shearing methodologies and avoiding the amplification step in the library preparation of the Ion Torrent PGM protocol. To test the fragmentation methods, LR-PCR products were sheared using the Covaris M220 sonicator (Life Technologies Europe, Gent, Belgium) and the NEBNext dsDNA Fragmentase (Bioke). For the first fragmentation method, a dilution to 100 ng in 50 µL of LR-PCR products were subjected to sonication for 130 s with a duty factor of 20%, a peak incident power of 50W, a temperature of 20°C and 200 cycles per burst, to tailor the DNA molecules into fragments with a median size of 200 bp (Ion Xpress Plus gDNA Fragment Library Preparation, Appendix B). A standard procedure was followed for the NEBNext dsDNA Fragmentase assay. Briefly, 1 µg of PCR product was added to 2 µL 10x Fragmentase reaction buffer and 0.2 µL of 100x BSA. This mixture was placed on ice for 5 min prior to the addition of 2 µL of NEBNext dsDNA Fragmentase and an incubation at 37°C for 30 min. The reaction was stopped by adding 5 µL of 0.5 M EDTA solution to the DNA fragments. Sheared samples were purified using AMPure beads. The size distribution of the fragmented DNA was assessed on the Bioanalyzer (Agilent, Diegem, Belgium), using the High Sensitivity Assay (Agilent, Diegem, Belgium). All further downstream manipulations were performed according to the Ion Torrent PGM protocol’s instructions (Ion Xpress Plus gDNA Fragment Library preparation, Life Technologies, Gent, Belgium). Briefly, samples were end repaired, ligated with adaptors, nick repaired and bead purified prior to amplification of size selected (E-gel system, Life Technologies) fragments around 330 bp long. Fragment sizes were assessed using the Bioanalyzer system and quantified with the Qubit 2.0 fluorimeter (Life Technologies, Gent, Belgium). Pooled libraries were used for emulsion PCR amplification. Sequencing reactions were run on the Ion Torrent PGM using Ion 316 version 2 chips and the Ion PGM 200 sequencing kit (Life Technologies, Gent, Belgium).

### Illumina MiSeq sequencing

To obtain 350 bp fragments LR-PCR products were sheared with the Covaris M220 sonicator (Life Technologies Europe, Gent, Belgium) and the NEBNext dsDNA Fragmentase enzyme (Bioke, Leiden, The Netherlands), both starting with 1 µg LR-PCR product. Covaris sheared LR-PCR products were fragmented using custom instrument specifications (TruSeq DNA PCR-Free Sample Preparation Guide). The protocol described, before concerning the NEBNext dsDNA Fragmentase, was the same except for the incubation time that was adapted to 15 min to obtain 350 bp fragments. Next, samples were further processed using the TruSeq DNA PCR-Free Sample Preparation protocol as instructed by the supplier (Illumina, Eindhoven, The Netherlands). After fragmentation, end repair, adenylation, and indexed paired end adapter ligation, samples were pooled and processed on the MiSeq sequencer with the MiSeq Reagent Micro Kit, v2 (Illumina). Conversely, all six samples were also processed using the Nextera XT kit (Illumina). A single Nextera tagmentation enzymatic reaction was used where LR-PCR products were simultaneously fragmented and tagged with adaptors. Finally, a limited cycle PCR protocol (12 cycles) was applied, adding simultaneously sequencing indexes (Nextera XT DNA Sample Preparation Guide, Illumina).

### Detection threshold determination for the MiSeq

The technical error rate of the MiSeq platform was determined with the methodology used for the Ion Torrent PGM system [4]. For the latter device, which unlike PhiX for the Illumina MiSeq lacks an endogenous control sample, a well typed pUC19 plasmid was used. The use of the same pUC19 DNA sample also allowed a comparison of sequencing results across platforms. One µg of pUC19 plasmid DNA (Thermo Fisher, Erembodegem-Aalst, Belgium) was sheared by the Covaris or NEBNext dsDNA Fragmentase. Subsequently, samples were processed using the TruSeq DNA PCR-Free Sample Preparation protocol, and sequenced on the MiSeq. The error rate of the sequencing process was computed by calculating the ratio of non-reference versus total bases per position. Taking the average of all ratios per position resulted in the average error rate of the pUC19 plasmid DNA.

### Data analysis

FastQ files from all datasets, generated by either the Ion Torrent PGM or MiSeq platforms, were mapped to the mitochondrial revised Cambridge Reference Sequence (rCRS, NC 012920.1) using BWA-MEM (version 0.7.5) [6]. As a metric for coverage bias, the relative coverage was used. Applying the SAMtools software (version 0.1.18) [7] the number of reads mapping to each reference base was counted. The mean coverage was calculated by averaging this value across each base in the sequence. By computing the ratio of the coverage of a given reference base and the mean coverage of all reference bases, the relative coverage was obtained. This was calculated for the plus and minus strand separately, for the total coverage of both strands together, and was presented in graphical illustrations. To visualize the relative coverage resulting from all different protocols and methods tested, circular plots were generated with the freeware Circos-0.64 software [8]. The Circos plots demonstrated in this article are restricted to sample 1, as the coverage profiles were consistent across all samples. To compare different methodologies, datasets were down sampled to an average coverage of 3000 using Picard (http://picard.sourceforge.net). The average relative coverage was collected for all samples processed with the same protocol resulting in seven datasets (Ion Torrent standard, Ion Torrent without amplification step, Ion Torrent Covaris, Ion Torrent NEBNext dsDNA Fragmentase, TruSeq Covaris, TruSeq NEBNext dsDNA Fragmentase and Nextera XT). For each dataset the fraction with a relative coverage <0.50; <0.25; <0.10; <0.05 and <0.01 was determined. To identify the nucleotide composition of undercovered regions GC, AT along with CT, AG, AC and GT dinucleotide motif plots were created and correlated to the total relative coverage, as well as the relative coverage from each strand separately. Both the incidence (in percentages) of the dinucleotide motifs in the mtDNA molecule, and the relative coverage were calculated in bins of 150 nucleotides and illustrated as bias plots.

For variant calling, three different strategies were employed and compared. First, all data were analyzed using an in-house pipeline based on GATK. FastQ files were aligned to the rCRS using BWA-MEM and sorted. Next, GATK realignment around indels and recalibration was performed. The GATK Unified Genotyper was used for variant calling, without at random down sampling of reads to reduce coverage. Subsequently, all variants with a quality score <400 were filtered from the vcf data. Second, all data were also analyzed using the CLC Genomics Workbench (version 6.0.5) against the rCRS. Only variants with an average quality score >25 were selected. A third and last strategy was only implemented on the Ion Torrent data. PGM files were mapped and variants were called using the Torrent Suite 4.2.

For each sample analyzed with the Ion Torrent PGM or MiSeq device, the sequencing error was determined for each position of the genome sequence, with exception of the true variants (versus rCRS) detected in each sample. The average sequencing error and their standard deviations were determined for these six samples. Potential low heteroplasmic variant levels were compared to these values and utilized as a reliable baseline (index) to reduce the false positive rate of the data [4].

## Results and Discussion

### Assessment of different PGM protocols

**Figure 1:**
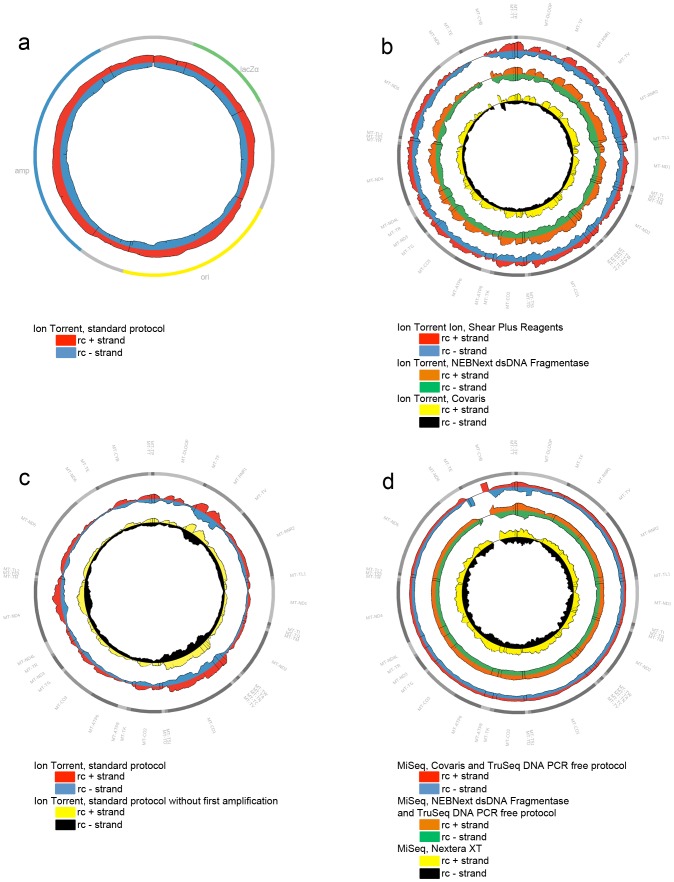
Genome Coverage plots. Representation of the MPS relative coverage of both strands (rc+: relative coverage of the plus strand, rc-: relative coverage of the negative strand) of the pUC19 plasmid, or mtDNA molecules obtained from the Ion Torrent PGM or MiSeq sequencing system. The outer circle symbolizes the pUC19 (A) or mtDNA (B, C, D) gene structure, respectively. **1A:** Use of the Ion Torrent PGM standard protocol on the pUC19 plasmid. **1B:** Use of three different fragmentation methods in combination with the Ion Torrent sequencing protocol on the mtDNA: Ion Shear Plus Reagents (enzymatic), NEBNext dsDNA Fragmentase (enzymatic) and Covaris (physical). **1C:** Use of an Ion Torrent PGM protocol without PCR amplification in the library construction on the mtDNA. **1D:** LR-PCR products of the mtDNA were Covaris (physical) or NEBNext dsDNA Fragmentase (enzymatic) sheared, followed by a TruSeq DNA PCR free protocol on a MiSeq instrument. The same six samples were processed with a Nextera XT kit (enzymatic shearing and PCR amplification in library preparation) prior to MiSeq analysis.

**Figure 2:**
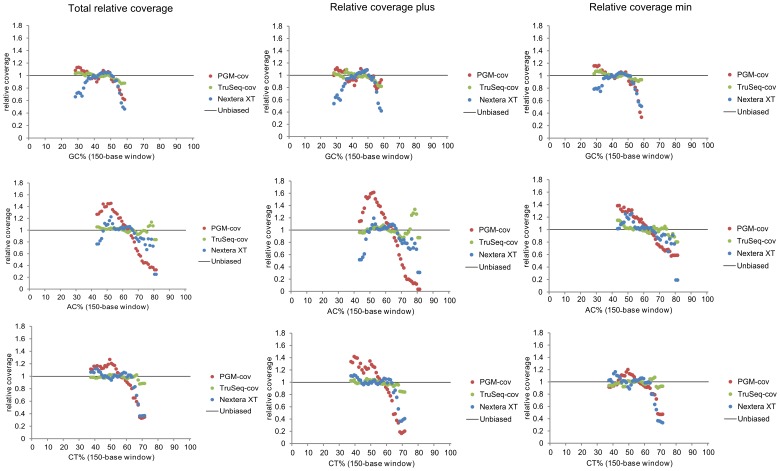
Nucleotide GC, AC and CT bias plots for the human mtDNA. The relative coverage as seen in this illustration is based on the average of the relative coverage of the six samples processed with the different protocols: Covaris shearing followed by the Ion Torrent protocol, Covaris shearing followed by the TruSeq procedure and the Nextera XT method. The average relative coverage was calculated for the total relative coverage and for both strand separately.

We have recently studied the use of the Ion Torrent PGM sequencer system in a diagnostic setting for the nucleotide analysis of human mitochondrial genomes of patient and control samples. The results uncovered a rather poor performance for some of the mtDNA regions [4]. Although it is well known that the PGM sequencing technology has problems handling homopolymeric stretches, an additional limitation was revealed, as a major difference in read depth between both strands was exposed for about 10% of the mitochondrial genome regions. For these sequences, the relative coverage of the positive strand dropped below 0.1. These particular patterns were reproduced in replicates of the same and between different samples, but never observed for pUC19 plasmid samples (Figure 1A). The causes of this remained unknown. Previous experiments had already excluded primer, LR-PCR or sample dependence, and it was assumed that the discrepancy originated from the enzymatic shearing step included in the Ion Torrent assay [4]. Altering fragmentation in the original Ion Torrent PGM assay could thus promote a change of the coverage profile. Hence, the standard enzymatic shearing step was omitted and substituted with an enzymatic treatment with NEBNext dsDNA Fragmentase or with physical shearing with a Covaris M220 sonicator device, leaving all further downstream process steps unchanged. Nonetheless, MPS data demonstrated that none of the altered protocols induced an equilibrated strand representation. Neither did they show an improvement of the under-representation of the plus strand. Uneven coverage was still produced (Figure 1B). Both shearing methods resulted still in 7 to 7.8% of the 16.2 kb fragment to have a relative coverage of the plus strand <0.1. Moreover, 2% of the 16.2 kb region showed a relative coverage of the plus strand <0.01 (Table 1). Further experiments, such as omission of the first PCR amplification step in the PGM library preparation protocol were carried out and subjected to MPS. But also this intervention did not lead to a reduced bias (Figure 1C, Table 1). By exchanging the Platinum Taq DNA polymerase for Kapa HiFi in the nick translation and amplification step during library preparation, Quail et al. [9] had demonstrated a reduced bias in PGM data. Therefore, it was proposed that the DNA amplification treatment during the library preparation and/or the emulsion PCR mediated a bias interfering with all further analysis of the mitochondrial genome. In order to further characterize the underlying mechanisms of poor PGM results across parts of the mitochondrial genome, the depth of the relative coverage seen at each position was tabulated for both strands separately. Hence, a possible association with its nucleotide composition was investigated systematically. In-house Perl scripts were used to calculate the content of GC or AT rich motifs, as well as any other dinucleotide rich combination. This analysis did not disclose any relationship between GC or AT rich regions and poor strand representation (Figure 2). The findings of Quail et al. [9] about very low coverage from GC or AT rich motifs for *P.falciparum* were not confirmed by the analysis of mtDNA. In contrast, reduced coverage was detected for AC and CT rich motifs. Particularly, coverage of the plus strand was negatively influenced by these two motifs. Moreover, the relative coverage of the plus strand dropped almost to zero for 80% (and more) AC rich motifs (Figure 2). The sequencing bias is seen for a high AC-content (range 70–80%) which corresponds to the figures of 80% and more for the GC and AT motifs presented by Ross et al. [1]. It is already known for a long time that the nucleotide composition of both mtDNA strands is different. The plus strand or *light* strand is C-rich, while the minus strand or *heavy* strand is G-rich. The rCRS is based on the L-strand and corresponds to the underrepresented plus strand in our sequencing results. The analyses were also performed for the pUC19 plasmid DNA. As expected, no correlation between its nucleotide composition and coverage data was observed (Supporting Information S2). We therefore hypothesize that the troughs generated by the Ion Torrent PGM system rather originate from the proliferation of the sheared mtDNA sequences and not from the fragmentation method *per se*. In fact, it might be inherent to the combination of the DNA polymerases used in the PCR amplification steps included in the standard protocols, and the nature of the mitochondrial genome sequence.

**Table 1:** Comparison between different methods and technologies based on relative coverage (RC) analysis of the data.

	Ion Torrent PGM											
RC	Standard			no library amplification			Covaris			NEBNext ds Fragmentase		
	Total	Plus	Min	Total	Plus	Min	Total	Plus	Min	Total	Plus	Min
<0.5	15.14	23.43	3.96	16.10	24.32	4.64	13.15	22.33	2.58	11.17	19.25	1.60
<0.25	1.36	13.12	0.02	1.10	13.73	0.09	0.88	12.70	0.35	0.27	11.52	0.29
<0.10	0.01	7.66	0.01	0.04	8.38	0.04	0.01	7.83	0.01	0.00	7.05	0.01
<0.05	0.00	5.47	0.01	0.00	6.02	0.01	0.00	5.81	0.01	0.00	4.95	0.00
<0.01	0.00	2.04	0.00	0.00	2.49	0.00	0.00	2.73	0.00	0.00	1.96	0.00
	**Illumina MiSeq**											
**RC**	**TruSeq-Covaris**			**TruSeq-NEBNext ds Fragmentase**			**Nextera XT**					
	**Total**	**Plus**	**Min**	**Total**	**Plus**	**Min**	**Total**	**Plus**	**Min**			
<0.5	0.27	1.56	1.47	0.39	1.64	1.14	7.03	9.20	9.57			
<0.25	0.01	0.81	0.83	0.01	0.73	0.42	1.64	2.61	2.48			
<0.10	0.01	0.38	0.35	0.01	0.12	0.02	0.01	0.26	0.01			
<0.05	0.00	0.23	0.20	0.00	0.01	0.00	0.01	0.06	0.01			
<0.01	0.00	0.00	0.00	0.00	0.00	0.00	0.00	0.00	0.00			

For all samples processed with a same protocol the average relative coverage was calculated and resulted in 7 different datasets. For each dataset, the fraction with a relative coverage <0.50, <0.25, <0.10, <0.05, <0.01 was determined. These analyses were performed for each strand separately (Plus, Min) and the total relative coverage (Total).

### Comparison PGM-MiSeq

We proceeded to study mitochondrial genome resequencing on a MiSeq platform, using two different strategies. The results of the PCR amplification free protocol of TruSeq were compared with those of the Nextera XT kit, a method including one PCR amplification step in the library preparation step. Experiments were carried out according to the manufacturer’s instructions. The average read depth for the different datasets generated with the MiSeq were 3723, 4701 and 19418 for the TruSeq Covaris, TruSeq NEBNext dsDNA Fragmentase and the Nextera XT methods, respectively. The reads generated by MiSeq (paired end reads), had a 150 bp fixed length, while reads generated by Ion Torrent PGM showed a variable single-end read length with an average of 145 bp. To compare different methodologies, datasets were down sampled to an average coverage of 3000. Relative coverage analysis showed a major improvement in strand equilibration for the TruSeq data. Data from the TruSeq sheared with the Covaris protocol, and the TruSeq enzymatically digested with NEBNext dsDNA Fragmentase achieved an impressive relative coverage, with few areas (only 1.6% and 1.6%, respectively) of the plus strand <0.5. The Nextera XT data did not show strand bias as seen with the PGM data. However, a general unevenness of coverage of both strands was seen. Indeed, regions of both strands (9.2% of the plus strand and 9.6% of min strand) showed a relative coverage <0.5. (Figure 1D, Table 1) which were associated with CT rich motifs. Unlike for the PGM, where mainly the positive strand was involved, both strands were affected, however not as severe as for the Ion Torrent data (Figure 2).

### Detection limit of the MiSeq

The detection threshold for the identification of base variants was set on 5% for the Ion Torrent chemistry. This value was based on the determination of the sequencing error and the sensitivity and specificity experiments previously performed [4]. To set the detection threshold for the MiSeq, the same pUC19 plasmid DNA sample was sheared with two different methods, once using the Covaris M220 sonicator and secondly using the NEBNext dsDNA Fragmentase. Both differentially sheared samples were sequenced on the MiSeq following TruSeq PCR free library preparation and a 100% coverage was obtained with an average read depth of 30 440 and 30 966 respectively. Similar average sequencing error results were obtained with 0.27% and 0.19% for the Covaris sheared sample and the enzymatic sheared sample respectively. These values are in concordance with the error rate obtained by the PhiX, which presented with an error rate of 0.35%. These error rates in turn correspond to previously reported data for the MiSeq platform [9]. By applying these results to determine the variant threshold for the mitochondrial resequencing, a detection threshold level of 2% is possible. However as the PGM data were previously investigated with a detection threshold of 5%, these settings were also used for the MiSeq data.

### Variant calling

Last, we assessed variant detection in all samples using the data panel of nucleotide alterations reflecting the Sanger sequencing previously performed. The majority of these variants were identified on both platforms (Table 2; Supporting Information S3). Results were collected for a PGM, TruSeq or Nextera XT dataset. Two variant calling pipelines, an in-house pipeline based on GATK and the Quality-based variant detection method (CLC Genomics Workbench) were applied to MiSeq datasets, and subsequently compared to the results of our previous study. The TS4.2 was only used with the PGM data. The first pipeline resulted in 99.5% of the variants detected in the TruSeq and Nextera XT dataset, while the PGM dataset showed a 92.4% concordance with the Sanger sequencing results. The CLC Genomics Workbench pipeline requires the variant to be present on both strands. 93.4%, 97.7% and 97.2% of the Sanger sequencing variants were called in the TruSeq Covaris, TruSeq NEBNext dsDNA Fragmentase and the Nextera XT dataset, respectively. Applying these terms to the PGM data resulted in 84.7% concordance with Sanger sequencing. However, omitting the strand parameter identified 95.2% of the variants for PGM data. These figures demonstrated clearly the effect of strand bias on variant calling for the PGM data. Indeed, 67 out of 98 false negative results were present on one strand only. An additional analysis with the TS4.2 software identified 96.6% of the variants. Three positions, m.294T>C, m.16183A>C and the polymorphic 302_316 region, presented as false negative results in the PGM data sets. An additional false negative variant, at position m.5899_5900insC escaped variant calling. All of these variants are situated near a homopolymeric stretch and, with the exception of m.5899_5900insC, are also located in regions with significant AC contents and its associated strand bias (relative coverage of the plus strand <0.2). It must be pointed out that, despite the well documented shortcoming in homopolymer calling, the propriety software is clearly well fitted for the PGM needs in variant calling. Comparing the various algorithms applied in this present and the previous study, the TS4.2 software was noticeably the better performer. Compared to the former TS3.6 version, a remarkable improvement was noticed for the false positive rate. Reanalyzing all PGM samples with the TS4.2 release showed a reduction in false positives from 13,4% to 8,9%, with a detection threshold level of 5%. The highest sensitivity for the MiSeq results (TruSeq and Nextera XT data) was obtained by our in-house pipeline based on GATK. Indeed, the only false negative result for these data was one specific variation in the polymorphic 302_316 region in sample 21. Two single nucleotide insertions were detected in this region with Sanger sequencing (m.309_310insC and m.315_316insC), but MiSeq identified them incorrectly as a heteroplasmic sequence mixture of molecules with an insertion of two or four C’s at position 309. Analysis of the Ion Torrent PGM data previously had revealed four variants (m.7989T>C, m.9769T>C, m.10866T>C, m.12071T>C) hitherto not identified by Sanger sequencing. These same variants were identified by the Illumina system with analogous allele frequencies (Table 3). In this project, a detection threshold of 5% was used for all data analysis. However, it must be pointed out that a more stringent detection threshold of 2% is possible for both the PGM and MiSeq data. From a diagnostic perspective these low detection limits are not always relevant. Most pathogenic mutations have a disease threshold well >60%. Nonetheless, in the context of genetic counseling of asymptomatic female relatives for family planning, low detection limits might be indicated. Adjusting the detection limit to 2% in our sample cohort identified two additional heteroplasmic variants on the MiSeq platform. A novel heteroplasmic variant m.8207C>T (p.(Pro208Ser)) in the *MT-CO2* gene was revealed in the mtDNA of leukocytes of patient 9. Another heteroplasmic variant, m.5609T>C, was identified in leukocytes of patient 14 in the *MT-TA* gene. Both allele frequencies, 2% and 4% respectively, were below the applied detection limit of the PGM sequencer. Both nucleotide variants, however, were acknowledged by the PGM data as well, as was indicated by review of the BAM files in IGV and reanalysis of the data using a detection threshold of 0.8% (corresponding to the sequencing error rate of the PGM device). It must be pointed out that, although the accuracy of low level heteroplasmy determination is heavily dependent on the depth of coverage, it is also defined by the sequencing error of the system. The latter being related to PCR, platform technologies, and the various algorithms implemented at the different steps of data processing.

**Table 2:** Comparison between the number of variants detected with the MPS and Sanger sequencing technologies.

		PGM^a^									MiSeq^b^									
Sample	Sangersequencing	Ion shearenzymes			Covaris			NEBNext dsDNAFragmentase			Covaris			NEBNext dsDNAFragmentase				Nextera XT		
		vs Sanger	FN	extra	vs Sanger	FN	extra	vs Sanger	FN	extra	vs Sanger	FN	extra	vs Sanger	FN		extra	vs Sanger	FN	extra
1	33	32	1	1	32	1	1	32	1	1	33	–	1	33		–	1	33	–	1
2	13	11	2	1	12	1	1	12	1	1	13	–	1	13		–	1	13	–	1
4	33	32	1	–	31	2	–	31	2	–	33	–	–	33		–	–	33	–	0
9	36	35	1	2	35	1	2	35	1	2	36	–	3	36		–	2	36	–	2
14	57	56	1	–	56	1	–	56	1	–	57	–	1	57		–	1	57	–	–
21	42	40	2	–	41	1	–	41	1	–	41	1	–	41		1	–	41	1	–
**Total**	**214**	**206**	**8**	**4**	**207**	**7**	**4**	**207**	**7**	**4**	**213**	**1**	**6**	**213**		**1**	**6**	**213**		

FN: false negative result, extra: additional low allele frequency variants identified compared to Sanger sequencing.

a Ion Torrent PGM results obtained with the TS4.2 software.

b MiSeq data obtained with the in-house GATK pipeline.

**Table 3:** Overview of the minor allele frequency (in %) of heteroplasmic variants detected in this study.

Sample	Variant	PGM					MiSeq				
		Ion shear	Covaris	NEBNext	average	stddev	Covaris	NEBNext	Nextera XT	average	stddev
1	m.12071T>C	12.2	12.2	12.1	12.2	0.1	12	12	23	15.7	6.4
2	m.7989T>C	17.1	14.7	14.1	15.3	1.6	19	19	13	17.0	3.5
9	m.9769T>C	9.7	9	8.5	9.1	0.6	8	8	8	8.0	0.0
9	m.10866T>C	7.3	6	6.4	6.6	0.7	7	9	7	7.7	1.2
9	m.8207C>T	1.5	1.3	1.5	1.4	0.1	2	1	2	1.7	0.6
14	m.5609T>C	4	8	4.8	5.6	2.1	4	4	4	4.0	0.0
14	m.7453G>A	52	55	53	53.3	1.5	53	54	56	54.3	1.5

### Conclusion

MPS analysis is a powerful tool able to simultaneously detect and quantify sequencing variants. However, diagnostic settings have high demands regarding accuracy of test results. A high sensitivity is crucial to avoid a misdiagnosis, while a low false positive rate is necessary to minimize additional Sanger sequencing work for confirmation of pathogenic discoveries. Our current findings have illustrated that MPS protocols demand a thorough evaluation of their data, and validation of the result files before a possible implementation as a diagnostic test should be considered. In many laboratories MPS analysis is now part of daily diagnostic work. Selecting an appropriate methodology for MPS projects envisioned deserves the necessary attention. Assessment of the nucleotide content of DNA samples to be analyzed proved here to be an essential parameter, among others, for evaluation of the performance of a sequencing methodology or technology. In our hands, the current Ion Torrent PGM standard assay, even with modifications, suffered from lack of coverage consistency of the L-strand of the human mitochondrial genome, making an evaluation of heteroplasmy in these underrepresented regions cumbersome. Comparison of the PGM and MiSeq Nextera XT data results with the MiSeq PCR free sequencing method suggest that coverage bias might be generated by the enzymes involved in the amplification rounds of the MPS processes. Indeed, the Nextera XT method, which included a PCR amplification step, produced also more variation in coverage than the samples processed with the TruSeq DNA PCR-Free Sample Preparation protocol. Nextera XT certainly reduced, but did not resolve the coverage inconsistency. Further modifications, such as the use of another DNA polymerase in both amplification steps of the standard PGM workflow (one in the library preparation, and another in the emulsion PCR) may lead to further improvements. However, this might be a complex process and beyond the time management and financial scope of this project. Consequently, at this very moment the TruSeq DNA PCR-Free Sample Preparation protocol on the MiSeq system might be the most appropriate technology to address low copy number mtDNA heteroplasmy adequately.

## Supporting Information

Supporting Information S1 **Overview experiments.**
(DOCX)

Supporting Information S2 **Nucleotide GC, AC and CT bias plots for the pUC19 plasmid.** pUC19 DNA was processed with the Standard Ion Torrent protocol, Covaris sheared followed by the TruSeq procedure and the Nextera XT method.
(TIF)

Supporting Information S3 **Overview of the variant calling results obtained by the TS4.2 software for the Ion Torrent data and our in-house pipeline based on GATK for all MiSeq data and Ion Torrent PGM data.** P: Ion Torrent PGM sequencing; T: TruSeq DNA PCR-Free Sample Preparation protocol; NXT: Nextera XT method; S: sample; Ion: Ion shear enzymes; C: Covaris; N: NEBNext dsDNA Fragmentase; TS4.2: analyzed with the Torrent Suite 4.2 software; GATK: analyzed with our in-house pipeline based on GATK.
(XLSX)
