# Supplementary material for: A Bumpy Ride on the Diagnostic Bench of Massive Parallel Sequencing, the Case of the Mitochondrial Genome
Source: PLoS One. 2014 Nov 10;9(11):e112950. doi: 10.1371/journal.pone.0112950 (PMC4226615; doi:10.1371/journal.pone.0112950)
Supplement: Supporting Information S1 — Overview experiments. (DOCX) [file pone.0112950.s001.docx]

Supporting Information S1 : Overview experiments

| Samples | Instrument | Protocol | Shearing method | LR-PCR* | Average coverage |
| --- | --- | --- | --- | --- | --- |
| 1 | Ion Torrent PGM | Ion Torrent standard | Ion | 3 | 6836 |
| 2 | Ion Torrent PGM | Ion Torrent standard | Ion | 3 | 5039 |
| 4 | Ion Torrent PGM | Ion Torrent standard | Ion | 3 | 6720 |
| 9 | Ion Torrent PGM | Ion Torrent standard | Ion | 3 | 7772 |
| 14 | Ion Torrent PGM | Ion Torrent standard | Ion | 3 | 7027 |
| 21 | Ion Torrent PGM | Ion Torrent standard | Ion | 3 | 6714 |
| 1 | Ion Torrent PGM | Ion Torrent standard | Ion | 1 | 5343 |
| 4 | Ion Torrent PGM | Ion Torrent standard | Ion | 1 | 7983 |
| 1 | Ion Torrent PGM | Ion Torrent no amplification | Ion | 3 | 3445 |
| 2 | Ion Torrent PGM | Ion Torrent no amplification | Ion | 3 | 4588 |
| 1 | Ion Torrent PGM | Ion Torrent | Covaris | 1 | 3948 |
| 2 | Ion Torrent PGM | Ion Torrent | Covaris | 1 | 4115 |
| 4 | Ion Torrent PGM | Ion Torrent | Covaris | 1 | 4192 |
| 9 | Ion Torrent PGM | Ion Torrent | Covaris | 1 | 4576 |
| 14 | Ion Torrent PGM | Ion Torrent | Covaris | 1 | 4187 |
| 21 | Ion Torrent PGM | Ion Torrent | Covaris | 1 | 4437 |
| 1 | Ion Torrent PGM | Ion Torrent | NEBNext ds Fragmentase | 1 | 4566 |
| 2 | Ion Torrent PGM | Ion Torrent | NEBNext ds Fragmentase | 1 | 6406 |
| 4 | Ion Torrent PGM | Ion Torrent | NEBNext ds Fragmentase | 1 | 4625 |
| 9 | Ion Torrent PGM | Ion Torrent | NEBNext ds Fragmentase | 1 | 5207 |
| 14 | Ion Torrent PGM | Ion Torrent | NEBNext ds Fragmentase | 1 | 4456 |
| 21 | Ion Torrent PGM | Ion Torrent | NEBNext ds Fragmentase | 1 | 5494 |
| 1 | Illumina MiSeq | Truseq DNA PCR-Free | Covaris | 1 | 3044 |
| 2 | Illumina MiSeq | Truseq DNA PCR-Free | Covaris | 1 | 5380 |
| 4 | Illumina MiSeq | Truseq DNA PCR-Free | Covaris | 1 | 3478 |
| 9 | Illumina MiSeq | Truseq DNA PCR-Free | Covaris | 1 | 3332 |
| 14 | Illumina MiSeq | Truseq DNA PCR-Free | Covaris | 1 | 3001 |
| 21 | Illumina MiSeq | Truseq DNA PCR-Free | Covaris | 1 | 4105 |
| 1 | Illumina MiSeq | Truseq DNA PCR-Free | NEBNext ds Fragmentase | 1 | 3659 |
| 2 | Illumina MiSeq | Truseq DNA PCR-Free | NEBNext ds Fragmentase | 1 | 4957 |
| 4 | Illumina MiSeq | Truseq DNA PCR-Free | NEBNext ds Fragmentase | 1 | 6333 |
| 9 | Illumina MiSeq | Truseq DNA PCR-Free | NEBNext ds Fragmentase | 1 | 1613 |
| 14 | Illumina MiSeq | Truseq DNA PCR-Free | NEBNext ds Fragmentase | 1 | 5533 |
| 21 | Illumina MiSeq | Truseq DNA PCR-Free | NEBNext ds Fragmentase | 1 | 6112 |
| 1 | Illumina MiSeq | Nextera XT |  | 1 | 13166 |
| 2 | Illumina MiSeq | Nextera XT |  | 1 | 8591 |
| 4 | Illumina MiSeq | Nextera XT |  | 1 | 11231 |
| 9 | Illumina MiSeq | Nextera XT |  | 1 | 57233 |
| 14 | Illumina MiSeq | Nextera XT |  | 1 | 15232 |
| 21 | Illumina MiSeq | Nextera XT |  | 1 | 11057 |

*LR-PCR: number of amplicons used in the long range PCR
