# Supplementary figures and images for: A Bumpy Ride on the Diagnostic Bench of Massive Parallel Sequencing, the Case of the Mitochondrial Genome
Source: PLoS One. 2014 Nov 10;9(11):e112950. doi: 10.1371/journal.pone.0112950 (PMC4226615; doi:10.1371/journal.pone.0112950)

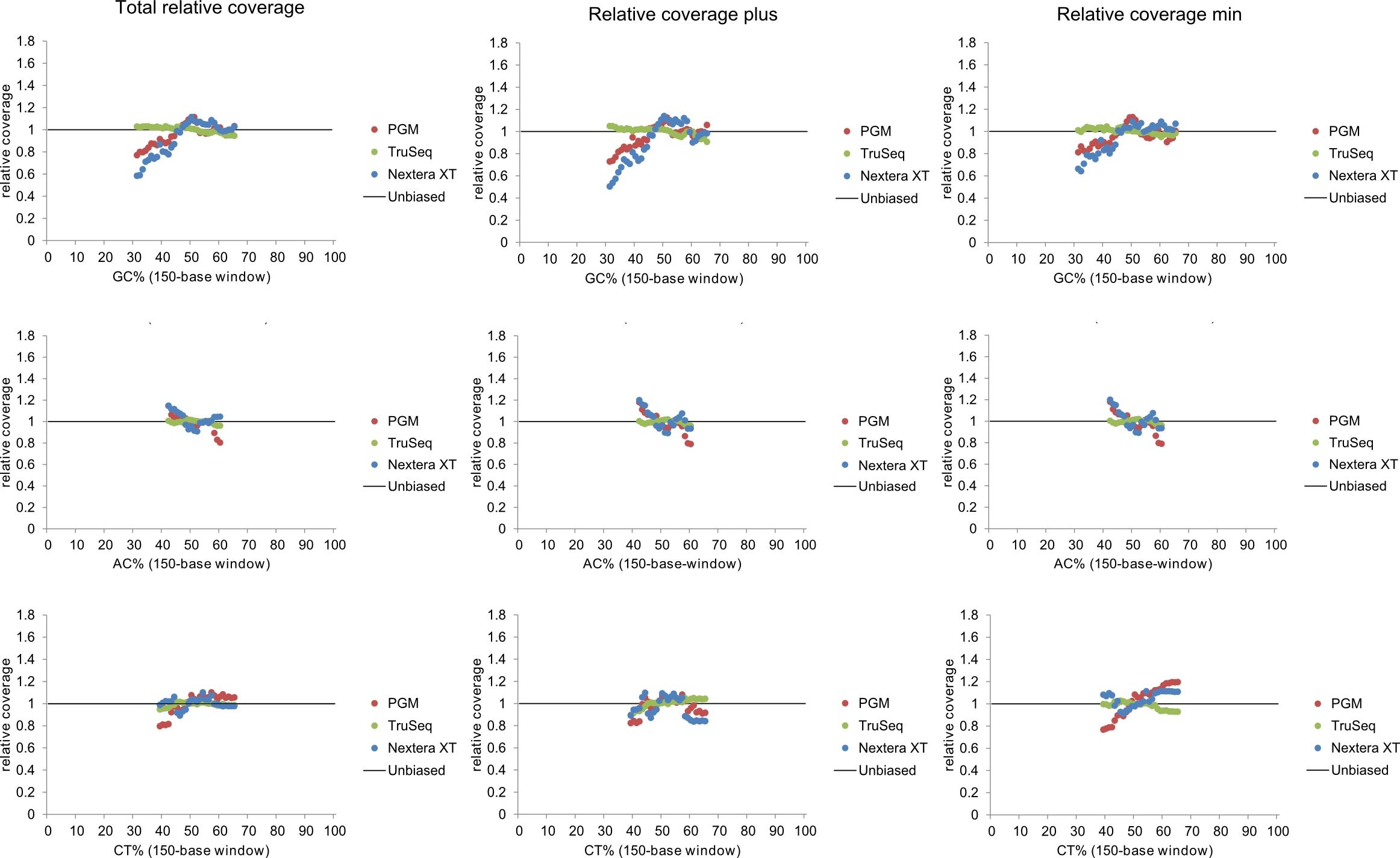

Supplement: Supporting Information S2 — Nucleotide GC, AC and CT bias plots for the pUC19 plasmid. pUC19 DNA was processed with the Standard Ion Torrent protocol, Covaris sheared followed by the TruSeq procedure and the Nextera XT method. (TIF) [file pone.0112950.s002.tif]
